# Chronic Low Dose Morphine Does Not Alter Two In Vitro BBB Models

**DOI:** 10.3390/brainsci12070888

**Published:** 2022-07-06

**Authors:** Jamie Marino, Monique E. Maubert, Jill M. Lawrence, Brian Wigdahl, Michael R. Nonnemacher

**Affiliations:** 1 Department of Microbiology and Immunology, Drexel University College of Medicine, Philadelphia, PA 19102, USA; jamie.marino21@gmail.com (J.M.); m.e.maubert@hotmail.com (M.E.M.); jl3785@drexel.edu (J.M.L.); bw45@drexel.edu (B.W.); 2 Center for Neurovirology and Translational Neuroscience, Institute for Molecular Medicine and Infectious Disease, Drexel University College of Medicine, Philadelphia, PA 19102, USA; 3 Sidney Kimmel Cancer Center, Thomas Jefferson University, Philadelphia, PA 19107, USA

**Keywords:** blood–brain barrier (BBB), transmigration, morphine

## Abstract

The blood–brain barrier (BBB) mediates cellular and molecular passage between the central nervous system (CNS) and peripheral circulation. Compromised BBB integrity has been linked to neurocognitive deficits in multiple diseases and various infections, including those associated with HIV-1 infection. Understanding the impact of exposure to pharmaceuticals, such as those utilized for pain management by patients suffering from CNS disease, on BBB regulation and function is clinically important. In this study, we modelled two different BBB systems; a primary human co-culture and a cell line monoculture. These systems were both exposed to three daily repeat doses of morphine and examined for alterations to BBB integrity via permeability, PBMC transmigration, and chemokine gradient changes. We did not find any significant changes to either BBB system with repeat morphine dosing, suggesting that repeat morphine exposure may not play a significant role in BBB changes.

## 1. Introduction

Despite the use of antiretroviral therapy (ART), people living with HIV-1 infections develop neurocognitive disorders at an accelerated rate compared to the general population [1,2]. These neurocognitive impairments are collectively referred to as HIV-1-associated neurocognitive disorders (HAND) and can vary in severity [1,2,3,4]. The development of HAND has been associated with blood–brain barrier (BBB) impairment, which can lead to increased inflammation and immune cell infiltration in the central nervous system (CNS), creating a chronic feedback loop of damage in the CNS and exacerbated HAND progression [5,6,7]. The BBB functions as a highly regulated and selective filter between the CNS and the peripheral circulation, mediating cellular and molecular passage between these biologic compartments [8,9]. The BBB is made up of specialized brain microvascular endothelial cells (BMECs) that are linked together by tight junction proteins (TJPs) and are supported by astrocytes [8,9,10]. Infected immune cells can cross the BBB to enter the CNS; therefore, the function and integrity of the BBB has been highlighted as a central component in the development of neurocognitive impairments [11,12].

Pain management therapies, such as morphine, a mu-opioid analgesic, are often prescribed to patients with neurocognitive impairments who experience chronic, painful symptoms [13,14,15]. In a study where a single dose of morphine (100 nM) was administered to primary human BMECs in the presence of HIV-1-infected peripheral blood mononuclear cells (PBMCs) there was downregulated mRNA expression of the TJPs, zonula occludens-1 (ZO-1) and occludin [13]. This study also found that one dose (24 h) of morphine caused an increase in BBB permeability, as measured by transepithelial electrical resistance (TEER) and enhanced HIV-1 infected PBMC transmigration across a co-culture model of the BBB using primary human BMECs and astrocytes [13]. In this study, we utilized daily morphine dosing (72 h) to mimic a treatment regimen and examine if the BBB is significantly impacted by morphine independently. Clinical concentrations of morphine in patients undergoing daily pain-therapy regimens, including those for cancers, have been reported to range from 40 nM to over 1000 nM in the blood, and from 1 nM up to 200 nM in the cerebrospinal fluid (CSF) [16]. Thus, the studies herein utilize morphine concentrations of 200 nM to reflect in vivo clinical concentrations of morphine observed upon daily exposure to this drug. We also chose to work in two different BBB model systems, to account for in vitro differences.

The first system utilizes a monoculture of the human brain microvascular endothelial cell line (hCMEC/D3) a human BMEC line that exhibits morphological and functional characteristics comparable to primary human BMECs, including contact inhibition and stable expression of normal endothelial markers such as the von Willebrand factor, cell adhesion molecules (CAMs), TJPs, adherens junctional proteins (AJPs), and chemokine receptors [17,18,19,20]. The benefit of using this model is the relative ease of acquiring and maintaining the cell line as opposed to the difficulty of obtaining primary human brain cells. Conversely, the disadvantage of an immortalized cell line is it may have, or develop, unknown alterations in normal cell functionality [19,20,21,22]. We have used this cell line previously and have shown that prolonged morphine exposure (up to 72 h) on hCMEC/D3 cell line cultures increased mRNA and protein expression of intracellular adhesion molecule-1 (ICAM-1), vascular cellular adhesion molecule-1 (VCAM-1), and activated leukocyte cell adhesion molecule (ALCAM), and enhanced firm adhesion of primary human PBMCs to the hCMEC/D3 cell line, which is a finding in contrast to the previously discussed study, and a contributing factor in the decision to use an additional BBB model system [13,22].

The second BBB model system utilizes a co-culture of primary human BMECs and astrocytes [1,4,23]. This system cultures BMECs and astrocytes on opposite sides of the inserts where BMECs have been shown to form junctional complexes and are in contact with astrocytes [24]. A benefit of this system is the utilization of two different primary human cells, which more closely mimics the BBB environment, rather than a system of BMECs alone. However, the disadvantage of this system is primary human cells can be difficult to acquire and can be passaged for a relatively short time, as compared to an immortalized cell line.

## 2. Materials and Methods

### 2.1. hCMEC/D3 Media, hCMEC/D3 Cell Line Culture, and In Vitro Blood-Brain Barrier Generation

Cells from the human brain microvascular endothelial cell line (hCMEC/D3, generously provided by Dr. Babette Weksler), were cultured in endothelial basal medium-2 (EBM-2) (Lonza, Quakertown, PA, USA) supplemented with heat-inactivated fetal bovine serum (FBS; FBS Gold; Sigma-Aldrich, Burlington, MA, USA), 1% penicillin-streptomycin (Corning, Corning, NY, USA), hydrocortisone (1.4 mM; Sigma-Aldrich, Burlington, MA, USA), ascorbic acid (5 mg/mL; Sigma-Aldrich, Burlington, MA, USA), 1% chemically defined lipid concentrate (Ceraplex; Thermo-Fisher Scientific Waltham, MA, USA), HEPES (10 mM; Corning, Corning, NY, USA), and basic fibroblast growth factor (bFGF; 1 ng/mL; Sigma-Aldrich, Burlington, MA, USA); referred to herein as complete hCMEC media. This formulation was previously validated and described by [25]. All experiments were performed between passages 27–32. Cells were grown on collagen-coated (Cultrex rat collagen I; R&D Systems, Minneapolis, MN, USA) 60 cm^2^ Petri dishes (TPP; Sigma-Aldrich, Burlington, MA, USA), 6-well plates (Corning, Corning, NY, USA), or 3 mm polycarbonate 3.8 cm^2^ 12-well transwell inserts with a pore size of 3.0 μm (Corning, Corning, NY, USA). The hCMEC/D3 cell line was seeded at a density of 37,000 cells/cm^2^ in Petri dishes and 6-well plates, and at 45,000 cells/cm^2^ in 12-well transwell inserts and grown to functional confluence prior to use in experimentation.

### 2.2. Primary Cell Culture

Primary human fetal astrocytes were obtained from the Temple University Comprehensive NeuroAIDS Center (CNAC) Core from 18-week aborted fetuses and subsequently cultured in DMEM, 10% heat inactivated fetal bovine serum (FBS; Gemini; Thermo-Fisher Scientific, Waltham, MA, USA), and 1% penicillin–streptomycin (Thermo Fisher Scientific, Waltham, MA, USA). Astrocytes were used between passage 2 to 6. Cells were grown on 60 cm^2^ Petri dishes (TPP; Sigma-Aldrich, Burlington, MA, USA), 24-well plates (Falcon, Corning, NY, USA), or 3 mm polycarbonate 0.3 cm^2^ 24-well transwell inserts with a pore size of 3.0 μm (Falcon, Corning, NY, USA). Astrocytes cells were seeded at a density of 100,000 cells/well in 24-well transwell inserts and grown to functional confluence prior to use in experimentation.

Primary human BMECs (Cell Systems, Kirkland, WA, USA) were cultured in Human M199 media (Invitrogen, Waltham, MA, USA), supplemented with 20% heat-inactivated newborn calf serum (Invitrogen, Waltham, MA, USA), 1% penicillin-streptomycin (Thermo Fisher Scientific, Waltham, MA, USA), 0.8% heparin (Sigma-Aldrich, Burlington, MA, USA), 5% heat-inactivated human serum AB (Corning, Corning, NY, USA), 0.1% ascorbic acid (Sigma-Aldrich, Burlington, MA, USA), 0.25% endothelial cell growth supplement (Sigma-Aldrich, Burlington, MA, USA), and 0.06% bovine brain extract (Lonza, Quakertown, PA, USA), referred to as M199C media. This media formulation is used with the human co-culture BBB model system, both of which were developed by [24] and validated in our laboratory [25]. Primary BMECs were used between passages 4–18. Cells were grown on 0.2% gelatin (Thermo Fisher Scientific, Waltham, MA, USA)-coated 60 cm^2^ Petri dishes (TPP; Sigma-Aldrich, Burlington, MA, USA), 24-well plates (Falcon, Corning, NY, USA), or 3 mm polycarbonate 0.3 cm^2^ 24-well transwell inserts with a pore size of 3.0 μm (Falcon, Corning, NY, USA). BMECs were seeded at a density of 40,000 cells/well in 24-well transwell inserts and grown to functional confluence prior to use in experimentation.

### 2.3. Human BBB Model

This model system was developed and characterized by [24] and validated in our laboratory. BMECs and human astrocytes were co-cultured on opposite sides of a gelatin-coated 3.0 μm pore tissue culture insert, according to previously published methods [1,4,26]. Astrocytes were first seeded on the basal side of the inserts, followed by BMEC seeding to the apical side. Cultures were maintained for three days in 24-well plates at 37 °C, CO_2_ (5%) in M199C media. At day three of co-culture, inserts were transferred to a low-serum version of M199C media (lacking human serum), until day four, at which time PBMCs were added to the apical chamber and allowed to transmigrate for 24 h. On day five, PBMCs and conditioned media were collected for flow cytometry and cytokine assays and the transwell inserts were analyzed for permeability.

### 2.4. Morphine and Other Reagents

Morphine sulfate salt pentahydrate, D-mannitol, and 70 kDa FITC-dextran were all obtained from Millipore Sigma, Burlington, MA. Flow cytometry antibodies were obtained from Abcam, Cambridge, UK (CD3, CD14) and Cell Signaling, Danvers, MA, USA (HRP-anti-rabbit secondary).

### 2.5. Morphine Exposure of Cells

At confluence, on 12-well (hCMEC/D3 cultures), or 24-well transwell inserts (primary BMEC-astrocyte co-cultures), cells were exposed to vehicle or 200 nM morphine for up to 72 h, with re-administration every 24 h.

### 2.6. Fluorescein Isothiocyanate—Dextran (FITC-D) Permeability Assay for hCMEC/D3

Monolayers of hCMEC/D3 cells were grown to functional confluence on collagen-coated 3.0 μM polycarbonate transwell inserts within six days, as previously determined by FITC-dextran permeability assays (Supplementary Figure S1). Inserts were exposed to morphine or 1.4 M mannitol (positive control for assay) for 30 min. At time zero, all chambers were washed with warmed 10 mM HEPES in 1X HBSS, followed by addition of unsupplemented EBM-2 media to bottom chambers and 2 mg/mL of 70 kD FITC-dextran (in unsupplemented EBM-2 media) to top chambers. At 5-min intervals up to 30 min, and at 60, 90, and 120 min, all bottom chambers were sampled, by transferring 100 μL to a white-walled optical-bottom 96-well plate. Fluorescence intensity was read on a Fluoroskan Ascent™ Microplate Fluorometer (Thermo Fisher Scientific, Waltham, MA, USA). The six time points at 5-min intervals were utilized to calculate permeability coefficients (Pe), or rate of passage through the monolayer, for each treatment condition.

Pe = PS/s, where PS (clearance) was the permeability surface area of the endothelial monolayer and s was the surface area of the filter (1.12 cm^2^). Pe is given as ×10^−5^ cm/s. PS was given by 1/PS = 1/me – 1/mf, where me and mf were the slopes of the curves corresponding to endothelial cells on filters and to filters only, respectively, with me and mf calculated by plotting the cleared volume against time. The cleared volume was calculated by (AUa–AUb)/Fi, where AUa was the total fluorescence (arbitrary units) in the basal compartment, AUb was the background fluorescence and Fi was the fluorescence of the initial solution (AU/mL).

### 2.7. BBB Permeability for Primary Co-Culture

Evans blue dye (0.5%; MilliporeSigma, Burlington, MA, USA) was added to 5% BSA and dissolved for 24 h at 4 °C to make Evans blue-coupled albumin (EBA), which is required for use in in vitro systems [26]. For the permeability assay, transwell inserts were first washed in phenol red-free DMEM (Thermo Fisher Scientific, Waltham, MA, USA). The inserts were transferred to 400 mL of 10% FBS/phenol red-free DMEM and 200 mL of 0.45% EBA was added to the apical side of the transwell. After a 30-min incubation the inserts were removed and discarded and the sample in the basal chamber was collected and read at 620 nm. For these studies, 4 nM and 0.5 M EDTA were used as positive controls for loss of barrier integrity, where 0.2 O.D. was determined to be the break point for barrier integrity.

### 2.8. CCL2 (MCP-1) ELISA

To determine the effects of morphine on the BBB in the hCMEC/D3 and primary co-culture systems, 24 h after PBMCs were added to the apical chamber the conditioned media was collected from the apical and basal chambers along with any PBMCs. The collected media was centrifuged to remove transmigrated cells and PBMCs were isolated from the basal chamber and used for flow cytometry while the supernatants from both the apical and basal chambers were assayed for CCL2 using the Human CCL2 ELISA procedure as described by the manufacturer (eBioscience, Thermo Fisher Scientific, Waltham, MA, USA). In these assays, stromal-derived factor (SDF-1) (CXCL12) was used as an additional positive control as it acts on both resting and activated T cells and can also be a chemoattractant for monocytes [24].

### 2.9. Transmigration/Flow Cytometry

Transmigration assays were performed on both model systems using PBMCs from healthy donors (Human Immunology Core, University of Pennsylvania, Philadelphia, PA, USA) added to the apical chambers at 300,000 cells/well for primary co-cultures and 750,000 cells/well for hCMEC/D3 cells. For positive control wells, 0.1% BSA in PBS (chemokine diluent), CCL2 (200 ng/mL; R&D Systems, Minneapolis, MN, USA), or CXCL12 (100 ng/mL; R&D Systems, Minneapolis, MN, USA) was added to the basal chamber. Transwells were incubated at 37 °C, 5% CO_2_ for 24 h to allow the PBMCs to transmigrate. After 24 h, the conditioned media and PBMCs were collected from both the apical and basal chambers and were centrifuged at 1200 rpm for 5 min at 4 °C to pellet the cells. The removed media was assayed for CCL2. The cells were washed twice in 1% BSA in PBS and centrifuged at 1200 rpm for 5 min at 4 °C. The appropriate antibody (CD3, CD14, live/dead) was added in 1% BSA in PBS and incubated on ice for 30 min in the dark. The cells were washed a second time in 1% BSA in PBS and centrifuged at 1200 rpm for 5 min at 4 °C. The wash was aspirated, and 2% paraformaldehyde was added to the cells. The prepared cells were then quantified by flow cytometry.

### 2.10. Statistics

Statistics for the FITC-Dextran assay were calculated using the ANOVA method, with log transformation and adjustment for possible effects of time, to compare the untreated to the morphine treatments and to mannitol. Statistics for other assays were calculated using the Student’s *t*-test. *p* values less than 0.05 were considered significant in all assays. Statistics were calculated utilizing Microsoft Excel and GraphPad Prism version 6.0.

## 3. Results

### 3.1. Morphine Exposure Does Not Alter the Basal Rate of Tracer Molecule Passage across hCMEC/D3 BBB Cells, Regardless of Single or Repeated Administration of Morphine

**Figure 1:**
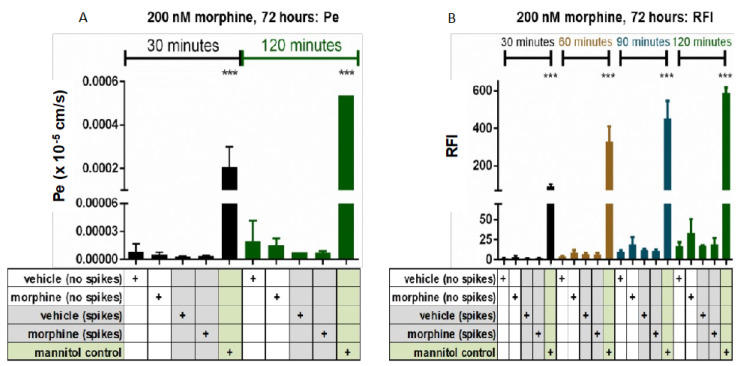
FITC-D Permeability of hCMEC/D3 transwell system. hCMEC/D3 cells cultured on collagen-coated polycarbonate (3.0 μM) transwell inserts were treated with vehicle (H_2_O) or morphine (200 nM), for a total of 72 h (**A**,**B**). Re-administration of vehicle or morphine (“spikes”) were performed every 24 h, with final re-administration conducted two hours before time zero, for all endpoints. Mannitol was incubated on positive control cells 30 min prior to time zero. Following exposure, all chambers were washed, and permeability was assessed by determining the amount of 70 kDa FITC-dextran to pass from the upper to lower chambers over 120 min. Permeability coefficient (Pe) (×10^−5^ cm/s) (**A**) and raw fluorescence intensity (RFI) (**B**) were calculated. All courses were performed in triplicate and are representative of three independent experiments. Statistical analysis was performed using the ANOVA method with log transformation and adjustment for possible time effects to compare the untreated to the morphine treatments and to treatment with mannitol. Based on 95% confidence intervals, no significant change was observed with morphine treatment. Mannitol treatment *** *p* < 0.0001.

Previous studies have reported differential outcomes of morphine exposure on BBB permeability to tracer molecule passage, depending on whether the system was in a state of constant repeated exposure versus withdrawal post-morphine exposure [22,27]. Here, we used single or repeated administrations of morphine at a concentration of 200 nM, to mimic chronic morphine exposure that would be given therapeutically to treat pain. We found that treatment with biologic concentrations of morphine at 200 nM, whether under single administration (“no spikes”) or repeated administration (“spikes”), did not increase baseline leakiness of the monolayer over 72 h, as compared to vehicle (H_2_O) controls (Figure 1).

### 3.2. Repeated, Prolonged Morphine Exposure Does Not Induce Cytokine Gradients across a hCMEC/D3 BBB Model

**Figure 2:**
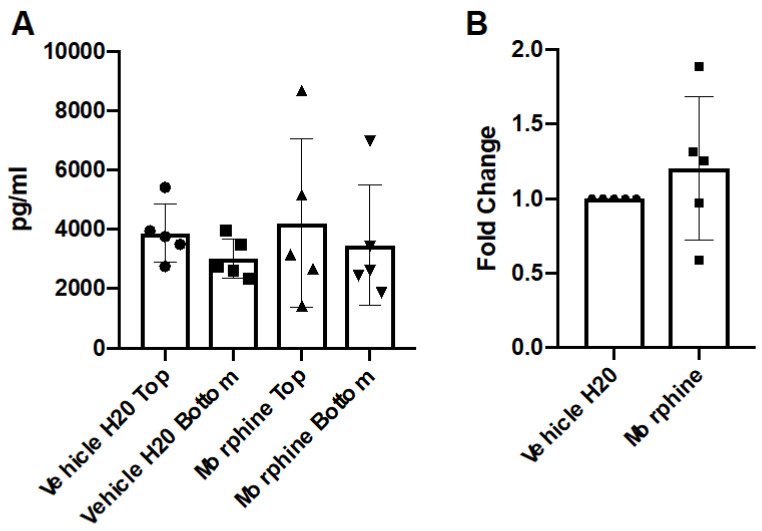
Concentration of CCL2 in hCMEC/D3 transwell system. hCMEC/D3 monolayers on collagen-coated 3.0 μM polycarbonate transwells were treated with vehicle (H_2_O) or morphine (200 nM) in complete hCMEC media for 72 h, with repeated administrations every 24 h. The final re-administration was performed two hours before the endpoint. Conditioned media from apical and basal chambers were harvested and assayed for CCL2 by ELISA. (**A**) The raw pg/mL values from the apical (TOP) chamber and the basal chamber (BOTTOM) are represented. (**B**) The fold change (pg/mL) of the basal chamber over the apical chamber was calculated and normalized to the vehicle (H_2_O) control. *n* = 5.

To address the potential role of morphine exposure in the generation of chemotactic gradients across the BBB, conditioned media from the apical and basal chambers were assayed for the expression of the cytokine CCL2 by ELISA. CCL2 was selected due to its recognized roles in the chemotaxis of monocytes in vitro and in vivo [26,28,29,30]. The results demonstrated that repeated administrations of morphine (200 nM) over 72 h exposures did not significantly alter the concentrations of CCL2 as compared to the vehicle control (Figure 2).

### 3.3. Repeated, Prolonged Morphine Exposure Does Not Alter PBMC Transmigration across a hCMEC/D3 BBB Model

**Figure 3:**
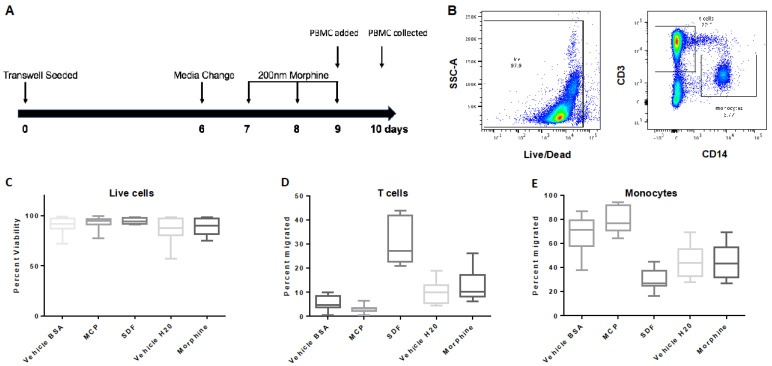
Transmigration of PBMCs across the hCMEC/D3 transwell system. (**A**) Map of experimental design where transwell inserts were seeded on day zero and grown to confluence until day six. Morphine (200 nM) was administered every 24 h for 72 h beginning on day seven. On day nine, morphine treatment was completed two hours before PBMCs were added to the apical chamber, and cells were allowed to transmigrate for 24 h before collection from the basal chamber on day 10. (**B**) PBMCs were stained for live/dead, CD3, CD14, and gated as shown. PBMCs were analyzed by flow cytometry and graphed as (**C**) live cells, (**D**) T cells, and (**E**) monocytes. Events (100,000 in number) were collected, +/− SD. Determined by student’s *t*-test. *n* = 5.

hCMEC/D3 cells were treated with either vehicle (H_2_O) or morphine (200 nM) every 24 h for 72 h (Figure 3A). To determine when the hCMEC/D3 monolayer reached confluence in the transwell, FITC-D permeability assays were performed and showed a significant reduction in FITC-D passage to the basal chamber on Day six that continued through to Day 10, as compared to the positive mannitol control (Supplementary Figure S1). At day nine, PBMCs were added to the apical chambers at a concentration of 1.5 × 10^6^ cells/mL and allowed to transmigrate for 24 h. We found that daily administrations of morphine (200 nM) over 72 h exposures did not significantly alter the transmigration of T cells or monocytes compared to the vehicle control (Figure 3C–E).

### 3.4. Morphine Exposure Does Not Alter Evans Blue Permeability across a Primary Co-Culture BBB Model

To determine a timeframe for when the primary co-culture reached confluence, we performed Evans blue permeability assays each day and found that cell confluence was established after day two (Supplementary Figure S2A). To ensure significant barrier integrity, EDTA at 4 mM and 0.5 M were also examined, which showed the consistent point where significant barrier impairment occurred was an O.D. of 0.2 (Supplementary Figure S2B). After 72 h of morphine treatment, as measured by Evans blue, there was no further increase in baseline leakiness of the co-culture with morphine treatment as compared to vehicle (H_2_O) (Supplementary Figure S3).

### 3.5. Repeated and Prolonged Morphine Exposure Does Not Induce Cytokine Gradients across a Primary Co-Culture BBB Model

**Figure 4:**
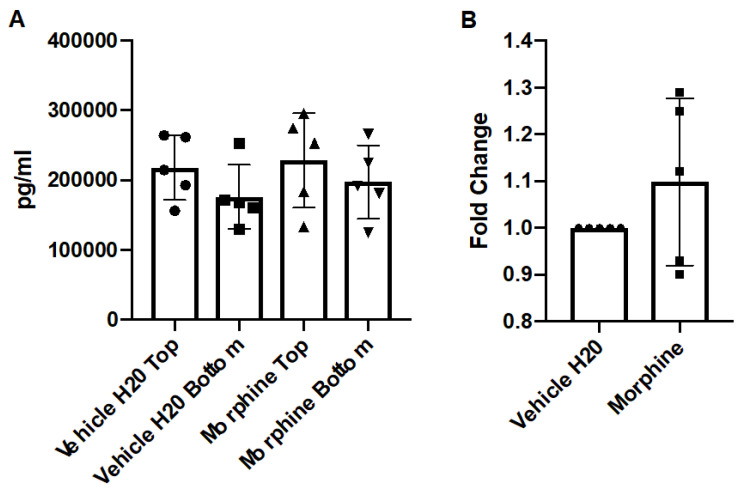
Concentration of CCL2 in a co-culture system. Primary human astrocytes and BMECs were seeded onto transwells and exposed to vehicle (H_2_O) or morphine (200 nM) in M199C media for 72 h, with repeated administrations every 24 h, with the final re-administration performed two hours before the endpoint. Conditioned media from apical and basal chambers were harvested and assayed for CCL2 by ELISA. (**A**) Graphed are the raw pg/mL values from the apical chambers (TOP) and basal chambers (BOTTOM) (**B**) The fold change (pg/mL) of the basal chamber over the apical chamber were calculated and normalized to the control. *n* = 5.

After morphine treatment, the media from the apical and basal chambers were assayed for expression of the cytokine, CCL2 by ELISA. The results demonstrated that repeated administrations of morphine over 72 h did not significantly alter concentrations of CCL2 compared to the control (Figure 4). These results were comparable to the results generated by the hCMEC/D3 model, with the caveat that the primary co-culture model generated much more concentrated levels of chemokine. However, the fold-change of each treatment of hCMEC/D3 to the primary co-culture model was comparable.

### 3.6. Repeated, Prolonged Morphine Exposure Does Not Alter PBMC Transmigration across a Primary Human Co-Culture BBB Model

**Figure 5:**
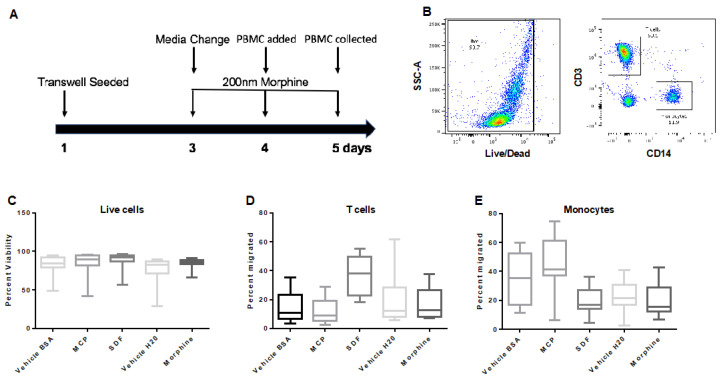
Transmigration of PBMCs across the primary co-culture transwell system. (**A**) Transwell inserts were seeded on day one, and morphine exposures (200 nM) were every 24 h for 72 h beginning on day three. On day four morphine was administered two hours before PBMCs were added, and cells were allowed to transmigrate for 24 h before collection from the basal chamber. (**B**) PBMCs were stained for live/dead, CD3, CD14, and gated, as shown. PBMCs were analyzed by flow cytometry and graphed as (**C**) live cells, (**D**) T cells, and (**E**) monocytes. Events (100,000 in number) were collected. +/− SD. Determined by student’s *t*-test. *n* = 5.

To examine how morphine may affect healthy PBMC transmigration across the BBB model, the co-culture was treated with vehicle (H_2_O) or morphine (200 nM) every 24 h for 72 h starting on day three (Figure 5A). On day four, healthy PBMCs were added to the apical chamber at a concentration of 1.5 × 10^6^ cells/mL and allowed to transmigrate for 24 h. After 24 h (day five), the PBMCs from the basal chamber were collected and stained for flow cytometry. The results demonstrated that repeated administrations of morphine over 72 h did not significantly alter transmigration of T cells or monocytes compared to the vehicle control (Figure 5C–E).

## 4. Discussion

This study examined the effects of daily morphine exposure in hCMEC/D3 cell line and primary human co-culture BBB models and found no overt alterations induced at the BBB in either model system. The concentrations of morphine used in this study mimic repeat morphine exposure that would be given therapeutically to treat chronic pain, where morphine administered in a hospital setting can result in concentrations of 250–350 nM in the blood. (https://www.wsp.wa.gov/breathtest/docs/webdms/DRE_Forms/Publications/drug/Human_Performance_Drug_Fact_Sheets-NHTSA.pdf; accessed on 14 June 2022).

In the hCMEC/D3 cell line model, we did not find increased baseline leakiness of the monolayer to small molecule passage, regardless of single versus repeated administrations of clinical doses of morphine. It has been shown that immortal BMECs do not have high TEER values, therefore we used FITC-D to measure permeability, as it has been supported in the literature for this model system [25]. In the primary co-culture system, we also did not find increased baseline leakiness of the barrier to Evans blue. Measuring permeability of the co-culture model by Evans blue has been previously established and validated by [24] as an appropriate method for this model system. Additionally, both FITC-D and Evans blue are colorimetric indicators, allowing for ease of comparison [28].

In the next set of experiments, we examined the production of a CCL2 chemokine gradient and the ability of this gradient to drive transmigration across the BBB. In the context of HIV-1 infection, there are elevated levels of pro-inflammatory cytokines in the CNS, which can recruit additional immune cells into the CNS, and lead to decreased BBB integrity [13,15,31,32]. An important chemokine involved in transmigration across the BBB is monocyte chemoattractant protein-1 (MCP-1; also referred to as chemokine C-C motif ligand 2, or CCL2), which is produced by many cell types including endothelial cells, fibroblasts, astrocytes, monocytes, and microglia [15,29,31]. CCL2 attracts monocytes, macrophages, T cells, NK cells, and dendritic cells to the site of interest and CCL2 gradients are capable of influencing BBB permeability by altered expression of TJPs, including occludin, claudin-5, and ZO-1 [22,31]. However, it was unknown if daily repeated morphine dosing that mimics a pain management regimen, is sufficient to create a chemokine gradient across a healthy BBB system. We observed no production of a CCL2 gradient across the chambers in either model system that was significant enough to alter transmigration of healthy PBMCs across the barrier. However, the concentration of CCL2 produced by the co-culture was much higher than that observed in the hCMEC/D3 cell line model, most likely due to the high production of CCL2 by astrocytes as compared to endothelial cells [33]. Additionally, the majority of PBMCs that did transmigrate across the transwells were alive, and the ratio of T cells to monocytes that transmigrated was also not significant.

We have previously shown that prolonged morphine exposure on hCMEC/D3 cells increased mRNA and protein expression of ICAM-1, VCAM-1, and ALCAM, and enhanced firm adhesion of PBMCs [22]. In this study, prolonged morphine exposure did not alter transmigration of healthy, uninfected PBMCs across either of the two different model systems. This might be explained if the PBMCs interact with the BBB due to the upregulation of adhesion molecules in hCMEC/D3 cells exposed to morphine; however, as there is no strong CCL2 chemokine gradient in these model systems, these cells may not be induced to cross the barrier into the respective basal chambers. Future experiments could explore this possibility by examining the amount of PBMCs adhered to the transwell in the presence and absence of morphine.

Throughout this study we did not find any significant differences between both model systems, and due to the difficulty in acquiring primary human cells, the immortalized cell line system may be a good option for preliminary experimentation of the BBB. A limitation of this study is that it only examined the effect of daily, repeat morphine exposure in the context of healthy PBMCs; however, it will be essential that future studies consider pathogenic conditions, such as HIV-1-positive PBMCs, which may interact with the BBB differently [34].

## 5. Conclusions

These studies have shown that repeat administration of clinically relevant concentrations of morphine does not significantly alter two different in vitro models of the BBB. In both the cell line monoculture of hCMEC/D3 cells and the primary co-culture of human BMECs and astrocytes, no changes in permeability, CCL2 concentrations, or PBMC transmigration after 72 h of daily morphine exposure at 200 nM was observed.

## Data Availability

Data is contained within the article or Supplementary Material.

## Supplementary Materials

The following supporting information can be downloaded at: https://www.mdpi.com/article/10.3390/brainsci12070888/s1, Figure S1: Time to confluent hCMEC/D3 barrier as measured by FITC-D.; Figure S2: Time to confluent co-culture as measured by Evans blue; Figure S3: Evans blue Permeability of co-culture transwell system.
