# Supplementary material for: Chronic Low Dose Morphine Does Not Alter Two In Vitro BBB Models
Source: Brain Sci. 2022 Jul 6;12(7):888. doi: 10.3390/brainsci12070888 (PMC9312884; doi:10.3390/brainsci12070888)
Supplement: Supplementary file 1 [file brainsci-12-00888-s001.zip › brainsci-1647002-supplementary.pdf]

# Chronic Low dose Morphine does not Alter Two In Vitro BBB Models

**Jamie Marino [1,2,†], Monique E. Maubert [1,2,†], Jill M. Lawrence [1,2], Brian Wigdahl [1,2,3] and Michael R. Nonnemacher [1,2,3,*]**

## Supplementary figures

**Supplemental Figure S1**

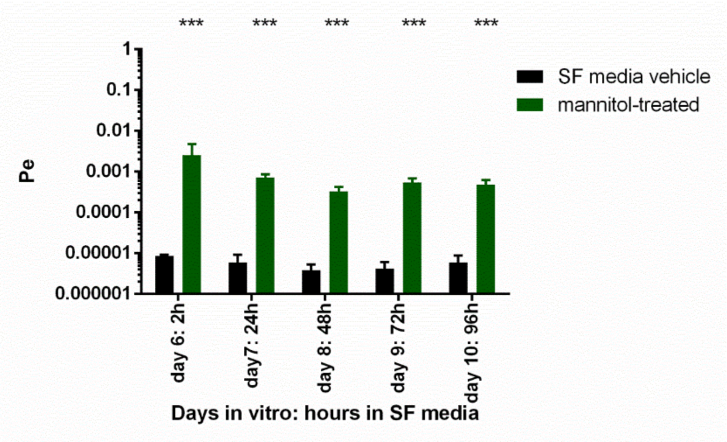


**Supplemental Figure S1**. **Time to confluent hCMEC/D3 barrier as measured by FITC-D**. hCMEC/D3 cells form a confluent barrier on polycarbonate transwells after six days and remain stably confluent up to 96 h in serum-free media. hCMEC/D3 cells cultured on collagen-coated 3 μM polycarbonate transwell inserts were cultured for six days in complete hCMEC media to functional confluence, then all chambers were washed twice with warmed 10 mM HEPES in 1X HBSS. Next, a media change with SF hCMEC media was performed. Cells were then cultured in serum free hCMEC media for a total of 2, 24, 48, 72, or 96 h. Mannitol was incubated on control cells 30 min prior to time zero. Following incubation with SF media, all chambers were washed, and permeability was assessed by determining the amount of 70 kDa FITC-dextran to pass from the apical to basal chambers over 30 min. Permeability coefficient (Pe) was calculated. Green bars indicate mannitol-treated inserts and black bars indicate serum-free media vehicle. All treatments were performed in triplicate and are representative of two independent experiments. Based on 95% confidence intervals no significant change was observed with any of the experimental exposure conditions (comparison of black bars). Mannitol treatment *** $p < 0.0001$

**Supplemental Figure S2**

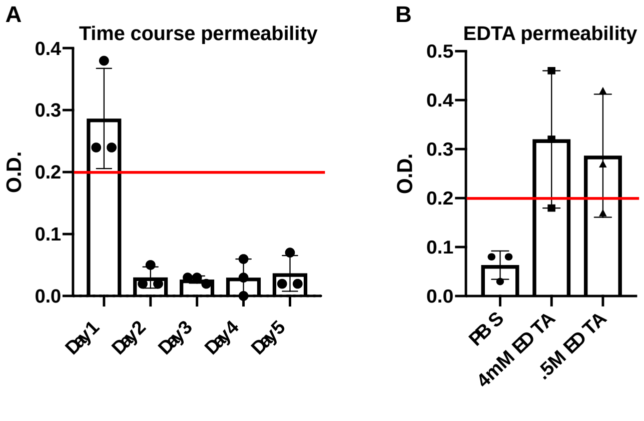


**Supplemental Figure S2. Time to confluent co-culture as measured by Evans blue.** Transwell inserts were seeded with astrocytes on the basal side and BMECs on the apical side at day one. (**A**) On days 1, 2, 3, 4, and 5, inserts were analyzed by Evans

blue to determine when the barrier reached confluence. (**B**) On day five, media in the apical and basal chambers was replaced with EDTA (4 mM or 0.5 M) for 30 min, prior to Evans blue permeability assay. Barrier break points were established as 0.2 O.D**.** The bars represent triplicates of individual experiments.

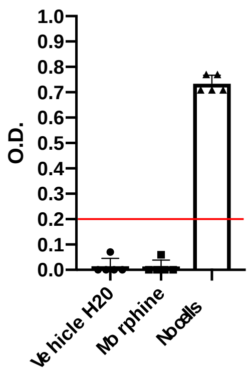


**Supplemental Figure S3. Evans blue permeability of co-culture transwell system.** Transwell inserts were seeded with astrocytes on the basal side and BMECs on the apical side at day one. Co-cultures were exposed to morphine (200 nM) every 24 h for 72 h beginning on day three. On day five, inserts were washed with PBS and Evans Blue was added to the apical chamber for 30 min, after which the apical chambers were removed, and the basal chamber was analyzed by spectrometry at 620 nm**.** 'No cells' indicates transwell alone. The break point was set to 0.2 O.D. units. $n$ = 5.
